# Supplementary material for: Task, person, and experiential characteristics drive the transfer of learning
Source: Commun Psychol. 2026 Jan 29;4:42. doi: 10.1038/s44271-026-00408-9 (PMC12957431; doi:10.1038/s44271-026-00408-9)
Supplement: Supplementary file 2 — Supplementary Materials [file 44271_2026_408_MOESM2_ESM.docx]

**SUPPLEMENTARY MATERIALS**

**Task, person, and experiential characteristics drive the transfer of learning**

Table of Contents

[Supplementary Methods 3](#_Toc214784994)

[Exploring Exponential vs. Power vs. Polynomial Learning Curves 3](#_Toc214784995)

[Supplementary Figure 1. Learning Curve Functional Forms. 4](#_Toc214784996)

[Supplementary Figure 2. Individual Transfer Trajectories. 5](#_Toc214784997)

[Supplementary Tables 6](#_Toc214784998)

[Table S1. Linear mixed effects model results predicting rounds 1–5 performance score and completion time (standardized relative to Round 1 – consistent – control) from training version, stress condition, round, and round squared. 6](#_Toc214784999)

[Table S2. Linear mixed effects model results predicting rounds 1–5 performance score and completion time (standardized relative to Round 1 – consistent – control) from training version, change in RMSSD relative to baseline, round, and round squared. 7](#_Toc214785000)

[Table S3. Linear mixed effects model results predicting rounds 1–5 performance score and completion time (standardized relative to Round 1 – consistent – control) from training version, change in MAP relative to baseline, round, and round squared. 8](#_Toc214785001)

[Table S4. Linear mixed effects model results predicting rounds 1–5 performance score and completion time (standardized relative to round 1 – consistent – control) from training version, change in HR relative to baseline, round, and round squared. 9](#_Toc214785002)

[Table S5. Linear mixed effects model results predicting rounds 5–6 performance score and completion time (standardized relative to round 1 – consistent – control) from training version, stress condition, round, and round 6 version switch. 10](#_Toc214785003)

[Table S6. Linear mixed effects model results predicting rounds 5-6 performance score and completion time (standardized relative to round 1 – consistent – control) from training version, change in RMSSD relative to baseline, round, and round 6 version switch. 11](#_Toc214785004)

[Table S7. Linear mixed effects model results predicting rounds 5–6 performance score and completion time (standardized relative to round 1 – consistent – control) from training version, change in MAP relative to baseline, round, and round 6 version switch. 12](#_Toc214785005)

[Table S8. Linear mixed effects model results predicting rounds 5–6 performance score and completion time (standardized relative to round 1 – consistent – control) from training version, change in HR relative to baseline, round, and round 6 version switch. 13](#_Toc214785006)

[Table S9. Linear mixed effects model results predicting rounds 1–5 performance score and completion time (standardized relative to round 1 – consistent – control) from emotion-cognition traits and their interactions with training version, stress condition, and round. 14](#_Toc214785007)

[Table S10. Linear mixed effects model results predicting rounds 1–5 performance score and completion time (standardized relative to round 1 – consistent – control) from emotion-cognition traits and their interactions with training version, change in RMSSD relative to base, and round. 16](#_Toc214785008)

[Table S11. Linear mixed effects model results predicting rounds 1–5 performance score and completion time (standardized relative to round 1 – consistent – control) from emotion-cognition traits and their interactions with training version, change in MAP relative to base, and round. 18](#_Toc214785009)

[Table S12. Linear mixed effects model results predicting rounds 1–5 performance score and completion time (standardized relative to round 1 – consistent – control) from emotion-cognition traits and their interactions with training version, change in HR relative to base, and round. 21](#_Toc214785010)

[Table S13. Linear mixed effects model results predicting rounds 5–6 performance score and completion time (standardized relative to round 1 – consistent – control) from emotion-cognition traits and their interactions with training version, stress condition, round, and round 6 version switch. 23](#_Toc214785011)

# Supplementary Methods

## Exploring Exponential vs. Power vs. Polynomial Learning Curves

Here, we report model-family checks for the trial-to-trial learning trajectory during acquisition (Rounds 1–5). The main text mixed-effects analyses used a polynomial basis (Round, Round²) with fixed effects of Stress (MAST vs. control) and Version (consistent vs. inconsistent mappings), and a participant-specific random intercept. To verify that our inferences were not an artifact of the chosen trajectory basis, we fit subject-level curves from three commonly used families: exponential $y=ae^{\lambda x}+c$, power $y=ax^{\lambda}+c$, and quadratic $y=ax^{2}+bx+c$. We did this with each participant’s series for two outcomes: standardized performance score and efficiency (time to complete a round, minutes). Participants with <5 observations were excluded. Fits used scipy.optimize.curve_fit with maxfev=100000; all models converged before hitting max iterations. Model quality was summarized per participant by Bayesian Information Criterion $BIC=nlog\left( \frac{RSS}{n} \right)+klog(n)$; lower BIC indicates better fit.

Results reproduced the mixed-effects conclusions using an entirely different fitting approach. For performance, the quadratic family yielded the lowest BIC distribution across participants (Total BIC_Quadratic_ = -2543.66; Mean BIC_Quadratic_ = -12.29), with the power model close behind and the worst fit being the exponential family (Total BIC_Power_ = -2216.89; Mean BIC_Power_ = -10.71; Total BIC_Exponential_ = -1846.82; Mean BIC_Exponential_ = -8.92). Visual inspection of fitted curves shows the expected monotonic, decelerating improvement that the quadratic basis captures well over five rounds. For completion time, the ordering shifted slightly: the power model produced the lowest BIC distribution, the quadratic was second, and exponential again under-performed (Total BIC_Quadratic_ = -4137.80; Mean BIC_Quadratic_ = -19.99; Total BIC_Power_ = -4830.47; Mean BIC_Power_ = -23.34; Total BIC_Exponential_ = -3548.24; Mean BIC_Exponential_ = -17.14). This pattern is consistent with multiplicative (percentage-like) gains in speed across rounds, versus more additive, saturating change in accuracy. Importantly, whether trajectories were parameterized as quadratic, power, or exponential did not change any qualitative inferences about Stress or Version from the mixed-effects models; the family choice mainly affected descriptive goodness-of-fit at the subject level.

**
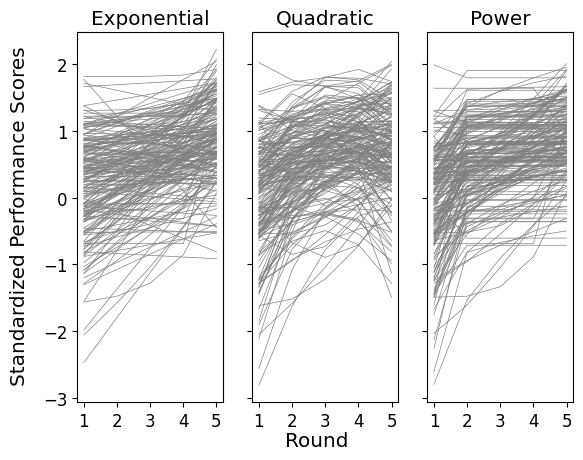

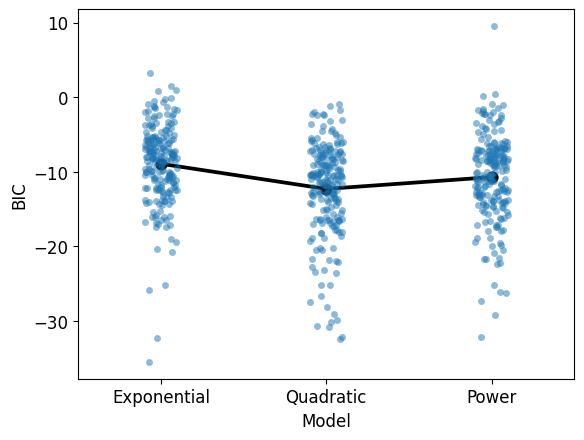
**

**
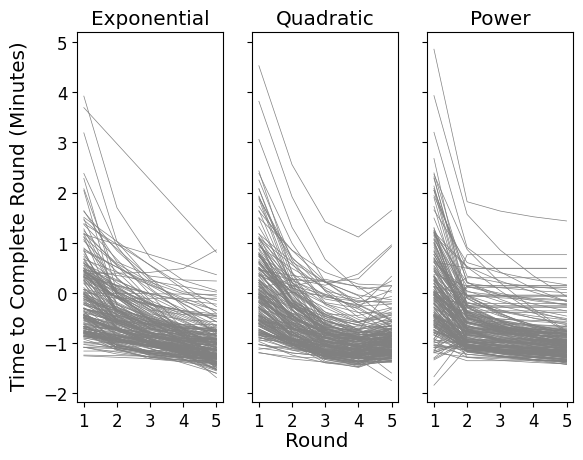

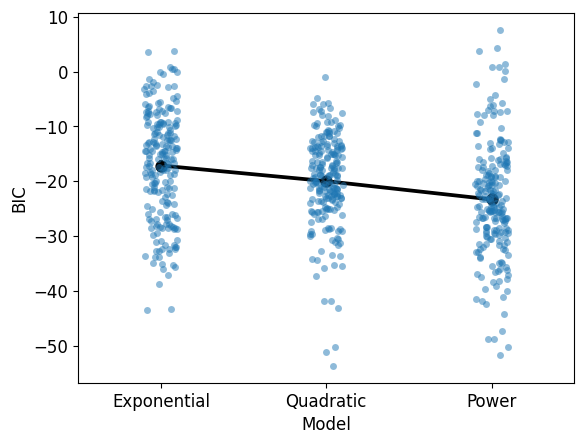
**

Supplementary Figure 1. Learning Curve Functional Forms. Top row (Performance): Left three panels show per-participant fitted curves (gray lines) for Exponential, Quadratic, and Power families; x-axis is Round (1–5), y-axis is Standardized Performance Score. Right panel shows per-participant BIC values (blue points) by family with the across-participant mean (black line). Lower BIC indicates better fit. Bottom row (Time): Same layout for Time to Complete Round (Minutes). Quadratic provides the best overall subject-level fit for performance; Power provides the best overall subject-level fit for time; Exponential is generally the worst fit for both outcomes. *N* = 241 participants.

**
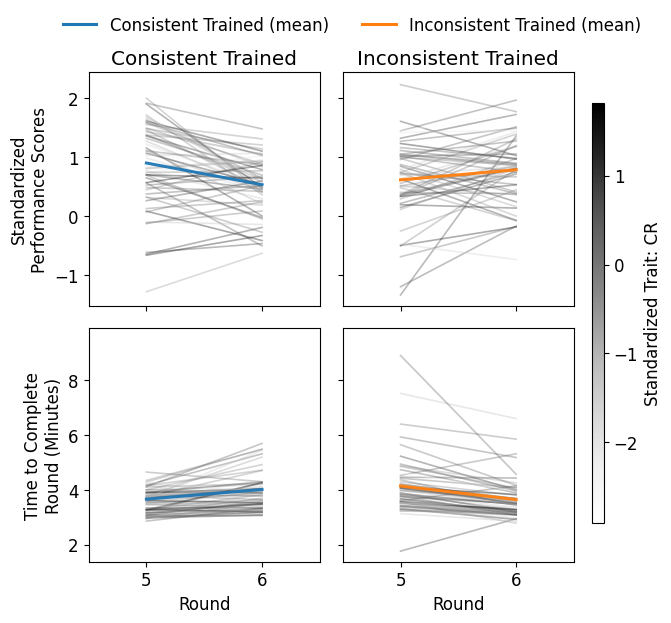

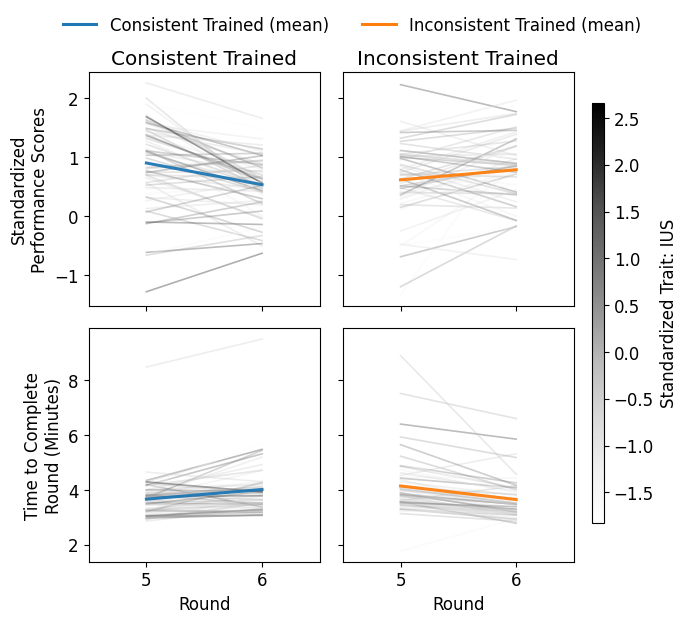
**

**
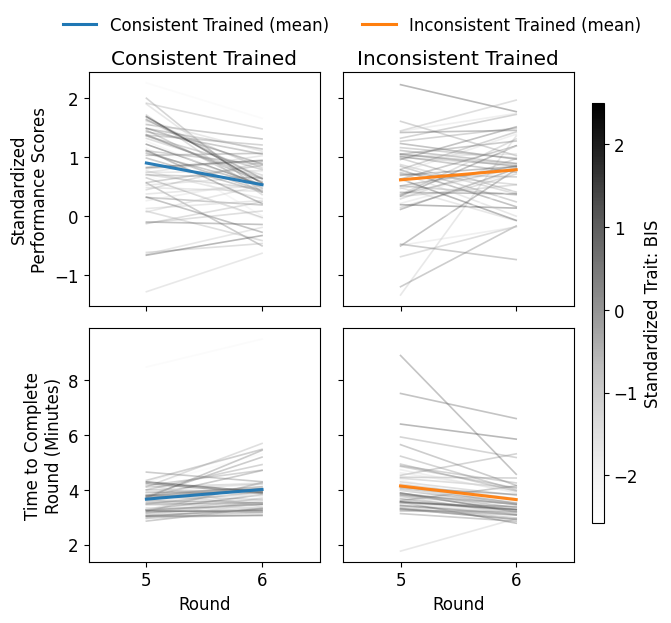

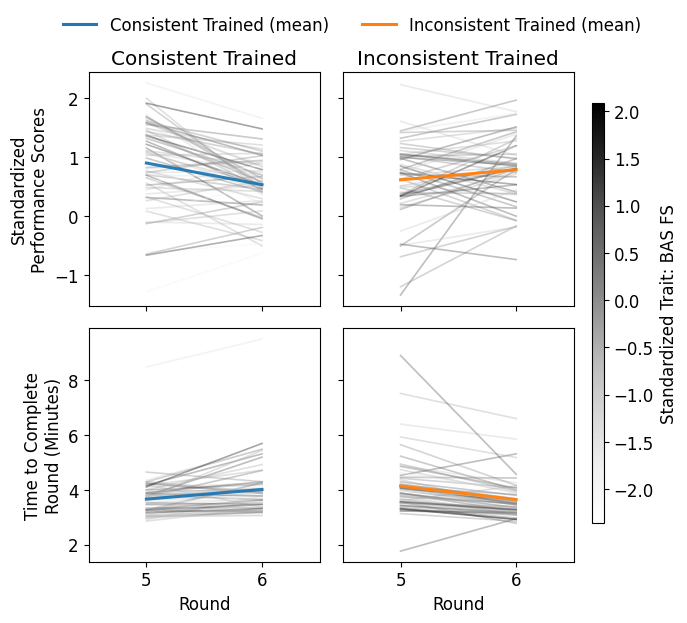
**

Supplementary Figure 2. Individual Transfer Trajectories. Post-switch change from Round 5 to 6 in performance (top) and completion time (bottom) by training version. Each gray line is one participant, with shade intensity proportional to a continuous trait (top left: cognitive reappraisal; top right: intolerance of uncertainty; bottom left: behavioral inhibition; bottom right: behavioral activation fun-seeking). Blue lines = average post-switch change for consistent trained, Orange lines = average post-switch change for inconsistent trained. *N* = 232 participants.

# Supplementary Tables

## Table S1. Linear mixed effects model results predicting Rounds 1–5 performance score and completion time (standardized relative to Round 1 – consistent – control) from Training Version, Stress Condition, Round, and Round Squared.

| Predictor | Performance Score | Completion Time |
| --- | --- | --- |
| Training Version (Inconsistent) | 0.029 (-0.212, 0.271) | 0.151 (-0.078, 0.379) |
| Condition (Stress) | 0.009 (-0.231, 0.250) | 0.006 (-0.222, 0.234) |
| Training Version (Inconsistent) × Condition (Stress) | -0.146 (-0.489, 0.197) | 0.120 (-0.205, 0.445) |
| Round | **0.348 (0.221, 0.476)***** | **-0.672 (-0.774, -0.570)***** |
| Training Version (Inconsistent) × Round | 0.081 (-0.102, 0.264) | 0.053 (-0.094, 0.199) |
| Condition (Stress) × Round | 0.174 (-0.009, 0.358) | 0.061 (-0.086, 0.207) |
| Training Version (Inconsistent) × Condition (Stress) × Round | -0.159 (-0.421, 0.104) | -0.096 (-0.306, 0.114) |
| Round Squared | **-0.043 (-0.073, -0.012)**** | **0.107 (0.082, 0.131)***** |
| Training Version (Inconsistent) × Round Squared | -0.032 (-0.076, 0.012) | -0.008 (-0.043, 0.027) |
| Condition (Stress) × Round Squared | -0.039 (-0.083, 0.005) | -0.012 (-0.047, 0.023) |
| Training Version (Inconsistent) × Condition (Stress) × Round Squared | 0.039 (-0.024, 0.103) | 0.011 (-0.040, 0.062) |

Note: Rounds 1–5 coded as 0–4. Standardized weights are reported alongside 95% confidence intervals in parentheses. * *p* < .05, ** *p* < .01, *** *p* < .001.

## Table S2. Linear mixed effects model results predicting Rounds 1–5 performance score and completion time (standardized relative to Round 1 – consistent – control) from Training Version, change in RMSSD relative to baseline, Round, and Round Squared.

| Predictor | Performance Score | Completion Time |
| --- | --- | --- |
| Training Version (Inconsistent) | -0.066 (-0.239, 0.107) | **0.201 (0.037, 0.365)*** |
| RMSSD Change | 0.126 (-0.032, 0.284) | 0.041 (-0.087, 0.169) |
| Training Version (Inconsistent) × RMSSD Change | -0.183 (-0.375, 0.009) | -0.092 (-0.247, 0.064) |
| Round | **0.417 (0.324, 0.511)***** | **-0.647 (-0.722, -0.572)***** |
| Training Version (Inconsistent) × Round | 0.024 (-0.109, 0.157) | 0.014 (-0.092, 0.121) |
| RMSSD Change × Round | -0.126 (-0.269, 0.017) | -0.008 (-0.123, 0.107) |
| Training Version (Inconsistent) × RMSSD Change × Round | 0.128 (-0.052, 0.308) | 0.067 (-0.077, 0.211) |
| Round Squared | **-0.059 (-0.081, -0.037)***** | **0.102 (0.084, 0.120)***** |
| Training Version (Inconsistent) × Round Squared | -0.016 (-0.048, 0.016) | -0.004 (-0.030, 0.021) |
| RMSSD Change × Round Squared | 0.026 (-0.004, 0.056) | 0.003 (-0.022, 0.027) |
| Training Version (Inconsistent) × RMSSD Change × Round Squared | -0.023 (-0.062, 0.015) | -0.013 (-0.044, 0.018) |

Note: Rounds 1–5 coded as 0–4. Standardized weights are reported alongside 95% confidence intervals in parentheses. * *p* < .05, ** *p* < .01, *** *p* < .001.

## Table S3. Linear mixed effects model results predicting Rounds 1–5 performance score and completion time (standardized relative to Round 1 – consistent – control) from Training Version, change in MAP relative to baseline, Round, and Round Squared.

| Predictor | Performance Score | Completion Time |
| --- | --- | --- |
| Training Version (Inconsistent) | -0.037 (-0.212, 0.138) | **0.174 (0.018, 0.329)*** |
| MAP Change | -0.043 (-0.136, 0.050) | 0.028 (-0.045, 0.102) |
| Training Version (Inconsistent) × MAP Change | 0.093 (-0.050, 0.236) | **0.143 (0.030, 0.257)*** |
| Round | **0.439 (0.346, 0.532)***** | **-0.649 (-0.721, -0.577)** |
| Training Version (Inconsistent) × Round | -0.017 (-0.151, 0.117) | -0.030 (-0.134, 0.075) |
| MAP Change × Round | 0.071 (-0.024, 0.166) | 0.001 (-0.073, 0.075) |
| Training Version (Inconsistent) × MAP Change × Round | -0.117 (-0.262, 0.029) | **-0.127 (-0.240, -0.013)*** |
| Round Squared | **-0.064 (-0.086, -0.041)***** | **0.102 (0.084, 0.119)***** |
| Training Version (Inconsistent) × Round Squared | -0.007 (-0.039, 0.025) | 0.006 (-0.019, 0.031) |
| MAP Change × Round Squared | -0.017 (-0.040, 0.006) | -0.002 (-0.019, 0.016) |
| Training Version (Inconsistent) × MAP Change × Round Squared | 0.024 (-0.011, 0.058) | 0.020 (-0.007, 0.047) |

Note: Rounds 1–5 coded as 0–4. Standardized weights are reported alongside 95% confidence intervals in parentheses. * *p* < .05, ** *p* < .01, *** *p* < .001.

## Table S4. Linear mixed effects model results predicting Rounds 1–5 performance score and completion time (standardized relative to round 1 – consistent – control) from Training Version, change in HR relative to baseline, Round, and Round Squared.

| Predictor | Performance Score | Completion Time |
| --- | --- | --- |
| Training Version (Inconsistent) | 0.005 (-0.174, 0.183) | **0.223 (0.055, 0.391)**** |
| HR Change | **0.141 (0.027, 0.255)*** | -0.026 (-0.123, 0.071) |
| Training Version (Inconsistent) × HR Change | **-0.156 (-0.310, -0.002)*** | -0.024 (-0.154, 0.107) |
| Round | **0.465 (0.371, 0.559)***** | **-0.648 (-0.724, -0.572)***** |
| Training Version (Inconsistent) × Round | -0.026 (-0.161, 0.108) | 0.002 (-0.107, 0.110) |
| HR Change × Round | -0.017 (-0.116, 0.081) | -0.006 (-0.085, 0.074) |
| Training Version (Inconsistent) × HR Change × Round | 0.074 (-0.069, 0.217) | -0.013 (-0.130, 0.103) |
| Round Squared | **-0.067 (-0.090, -0.045)***** | **0.102 (0.084, 0.120)***** |
| Training Version (Inconsistent) × Round Squared | -0.009 (-0.041, 0.023) | -0.003 (-0.028, 0.023) |
| HR Change × Round Squared | -0.006 (-0.029, 0.016) | 0.003 (-0.015, 0.022) |
| Training Version (Inconsistent) × HR Change × Round Squared | -0.013 (-0.046, 0.021) | 0.004 (-0.023, 0.031) |

Note: Rounds 1–5 coded as 0–4. Standardized weights are reported alongside 95% confidence intervals in parentheses. * *p* < .05, ** *p* < .01, *** *p* < .001.

## Table S5. Linear mixed effects model results predicting Rounds 5–6 performance score and completion time (standardized relative to round 1 – consistent – control) from Training Version, Stress Condition, Round, and Round 6 Version Switch.

| Predictor | Performance Score | Completion Time |
| --- | --- | --- |
| Training Version (Inconsistent) | -0.213 (0.541, 0.115) | 0.204 (-0.015, 0.424) |
| Condition (Stress) | 0.050 (-0.272, 0.371) | 0.176 (-0.037, 0.389) |
| Switch | 0.084 (-0.223, 0.391) | 0.029 (-0.177, 0.236) |
| Training Version (Inconsistent) × Condition (Stress) | -0.057 (-0.531, 0.417) | -0.191 (-0.504, 0.121) |
| Training Version (Inconsistent) × Switch | 0.043 (-0.409, 0.496) | -0.037 (-0.340, 0.265) |
| Condition (Stress) × Switch | -0.004 (-0.449, 0.441) | -0.193 (-0.490, 0.103) |
| Training Version (Inconsistent) × Condition (Stress) × Switch | -0.130 (-0.779, 0.519) | 0.277 (-0.154, 0.708) |
| Round | 0.047 (-0.154, 0.247) | -0.089 (-0.183, 0.005) |
| Training Version (Inconsistent) × Round | 0.131 (-0.166, 0.428) | 0.030 (-0.110, 0.169) |
| Condition (Stress) × Round | -0.059 (-0.358, 0.240) | -0.044 (-0.185, 0.098) |
| Switch × Round | **-0.386 (-0.665, -0.108)**** | **0.242 (0.111, 0.372)***** |
| Training Version (Inconsistent) × Condition (Stress) × Round | 0.065 (-0.371, 0.501) | 0..044 (-0.162, 0.250) |
| Training Version (Inconsistent) × Switch × Round | 0.300 (-0.115, 0.716) | **-0.373 (-0.568, -0.177)***** |
| Condition (Stress) × Switch × Round | 0.045 (-0.366, 0.456) | 0.041 (-0.153, 0.235) |
| Training Version (Inconsistent) × Condition (Stress) × Switch × Round | 0.095 (-0.505, 0.696) | -0.140 (-0.423, 0.144) |

Note: Standardized weights are reported alongside 95% confidence intervals in parentheses. * *p* < .05, ** *p* < .01, *** *p* < .001.

## Table S6. Linear mixed effects model results predicting Rounds 5-6 performance score and completion time (standardized relative to round 1 – consistent – control) from Training Version, change in RMSSD relative to baseline, Round, and Round 6 Version Switch.

| Predictor | Performance Score | Completion Time |
| --- | --- | --- |
| Training Version (Inconsistent) | -0.142 (-0.390, 0.105) | 0.146 (-0.015, 0.307) |
| RMSSD Change | -0.055 (-0.431, 0.321) | **-0.264 (-0.478, -0.049)*** |
| Switch | 0.067 (-0.157, 0.291) | -0.041 (-0.191, 0.108) |
| Training Version (Inconsistent) × RMSSD Change | 0.622 (-0.010, 1.254) | **0.395 (0.056, 0.734)*** |
| Training Version (Inconsistent) × Switch | -0.103 (-0.435, 0.230) | 0.057 (-0.162, 0.277) |
| RMSSD Change × Switch | -0.116 (-0.613, 0.381) | **0.307 (0.024, 0.590)*** |
| Training Version (Inconsistent) × RMSSD Change × Switch | -0.671 (-1.404, 0.063) | **-0.539 (-0.940, -0.138)**** |
| Round | 0.026 (-0.123, 0.176) | **-0.092 (-0.162, -0.022)*** |
| Training Version (Inconsistent) × Round | 0.060 (-0.171, 0.291) | 0.016 (-0.093, 0.125) |
| RMSSD Change × Round | 0.105 (-0.256, 0.467) | **0.196 (0.025, 0.367)*** |
| Switch × Round | **-0.354 (-0.561, -0.147)**** | **0.238 (0.141, 0.336)***** |
| Training Version (Inconsistent) × RMSSD Change × Round | **-0.712 (-1.344, -0.080)*** | -0.268 (-0.574, 0.038) |
| Training Version (Inconsistent) × Switch × Round | **0.444 (0.134, 0.754)**** | **-0.396 (-0.542, -0.250)***** |
| RMSSD Change × Switch × Round | -0.051 (-0.542, 0.439) | -0.208 (-0.439, 0.023) |
| Training Version (Inconsistent) × RMSSD Change × Switch × Round | **0.833 (0.080, 1.586)*** | **0.402 (0.040, 0.764)*** |

Note: Standardized weights are reported alongside 95% confidence intervals in parentheses. * *p* < .05, ** *p* < .01, *** *p* < .001.

## Table S7. Linear mixed effects model results predicting Rounds 5–6 performance score and completion time (standardized relative to round 1 – consistent – control) from Training Version, change in MAP relative to baseline, Round, and Round 6 Version Switch.

| Predictor | Performance Score | Completion Time |
| --- | --- | --- |
| Training Version (Inconsistent) | **-0.241 (-0.477, -0.005)*** | 0.107 (-0.052, 0.265) |
| MAP Change | -0.015 (-0.158, 0.128) | 0.024 (-0.053, 0.101) |
| Switch | 0.073 (-0.150, 0.297) | -0.059 (-0.211, 0.092) |
| Training Version (Inconsistent) × MAP Change | 0.127 (-0.080, 0.334) | -0.029 (-0.140, 0.081) |
| Training Version (Inconsistent) × Switch | 0.013 (-0.313, 0.340) | 0.091 (-0.129, 0.311) |
| MAP Change × Switch | 0.005 (-0.185, 0.196) | -0.010 (-0.111, 0.092) |
| Training Version (Inconsistent) × MAP Change × Switch | -0.195 (-0.479, 0.089) | -0.013 (-0.165, 0.139) |
| Round | 0.036 (-0.118, 0.190) | **-0.112 (-0.186, -0.039)**** |
| Training Version (Inconsistent) × Round | 0.122 (-0.104, 0.348) | 0.062 (-0.046, 0.171) |
| MAP Change × Round | 0.011 (-0.151, 0.174) | -0.018 (-0.097, 0.061) |
| Switch × Round | **-0.384 (-0.598, -0.169)***** | **0.258 (0.156, 0.360)***** |
| Training Version (Inconsistent) × MAP Change × Round | -0.062 (-0.295, 0.171) | -0.004 (-0.118, 0.109) |
| Training Version (Inconsistent) × Switch × Round | **0.369 (0.053, 0.685)*** | **-0.453 (-0.603, -0.302)***** |
| MAP Change × Switch × Round | 0.013 (-0.209, 0.235) | 0.032 (-0.076, 0.141) |
| Training Version (Inconsistent) × MAP Change × Switch × Round | 0.100 (-0.227, 0.426) | 0.023 (-0.137, 0.182) |

Note: Standardized weights are reported alongside 95% confidence intervals in parentheses. * *p* < .05, ** *p* < .01, *** *p* < .001.

## Table S8. Linear mixed effects model results predicting Rounds 5–6 performance score and completion time (standardized relative to round 1 – consistent – control) from Training Version, change in HR relative to baseline, Round, and Round 6 Version Switch.

| Predictor | Performance Score | Completion Time |
| --- | --- | --- |
| Training Version (Inconsistent) | -0.236 (-0.478, 0.006) | 0.083 (-0.075, 0.241) |
| HR Change | -0.034 (-0.188, 0.120) | 0.074 (-0.022, 0.171) |
| Switch | 0.095 (-0.134, 0.324) | -0.096 (-0.247, 0.055) |
| Training Version (Inconsistent) × HR Change | -0.081 (-0.312, 0.150) | **-0.147 (-0.289, -0.005)*** |
| Training Version (Inconsistent) × Switch | -0.039 (-0.371, 0.294) | 0.153 (-0.066, 0.372) |
| HR Change × Switch | 0.040 (-0.174, 0.254) | -0.111 (-0.245, 0.022) |
| Training Version (Inconsistent) × HR Change × Switch | 0.042 (-0.286, 0.370) | 0.254 (0.052, 0.457) |
| Round | 0.021 (-0.135, 0.177) | **-0.125 (-0.199, -0.051)*** |
| Training Version (Inconsistent) × Round | 0.159 (-0.063, 0.381) | 0.067 (-0.038, 0.172) |
| HR Change × Round | -0.009 (-0.161, 0.143) | -0.040 (-0.113, 0.032) |
| Switch × Round | **-0.367 (-0.578, -0.155)**** | **0.270 (0.170, 0.370)***** |
| Training Version (Inconsistent) × HR Change × Round | 0.074 (-0.150, 0.299) | 0.102 (-0.004, 0.209) |
| Training Version (Inconsistent) × Switch × Round | **0.387 (0.080, 0.695)*** | **-0.470 (-0.615, -0.324)***** |
| HR Change × Switch × Round | 0.006 (-0.201, 0.213) | 0.021 (-0.077, 0.119) |
| Training Version (Inconsistent) × HR Change × Switch × Round | 0.036 (-0.276, 0.347) | -0.145 (-0.293, 0.002) |

Note: Standardized weights are reported alongside 95% confidence intervals in parentheses. * *p* < .05, ** *p* < .01, *** *p* < .001.

## Table S9. Linear mixed effects model results predicting Rounds 1–5 performance score and completion time (standardized relative to round 1 – consistent – control) from emotion-cognition traits and their interactions with Training Version, Stress Condition, and Round.

| Predictor | Performance Score | Completion Time |
| --- | --- | --- |
| Training Version (Inconsistent) | 0.064 (-0.163, 0.29) | 0.199 (-0.02, 0.417) |
| Condition (Stress) | 0.109 (-0.124, 0.342) | 0.036 (-0.189, 0.26) |
| Training Version (Inconsistent) × Condition (Stress) | -0.194 (-0.527, 0.139) | 0.106 (-0.215, 0.427) |
| Round | **0.174 (0.136, 0.212)***** | **-0.239 (-0.272, -0.206)***** |
| Training Version (Inconsistent) × Round | -0.04 (-0.094, 0.014) | 0.011 (-0.036, 0.058) |
| Condition (Stress) × Round | 0.02 (-0.034, 0.075) | 0.004 (-0.043, 0.052) |
| Training Version (Inconsistent) × Condition (Stress) × Round | -0.003 (-0.081, 0.075) | -0.042 (-0.11, 0.026) |
| Cognitive Reappraisal | -0.054 (-0.199, 0.091) | -0.096 (-0.239, 0.048) |
| Cognitive Reappraisal × Training Version (Inconsistent) | -0.142 (-0.36, 0.075) | 0.047 (-0.168, 0.262) |
| Cognitive Reappraisal × Condition (Stress) | 0.063 (-0.156, 0.282) | 0.109 (-0.107, 0.325) |
| Cognitive Reappraisal × Training Version (Inconsistent) × Condition (Stress) | 0.062 (-0.26, 0.384) | 0.106 (-0.211, 0.423) |
| Expressive Suppression | 0 (-0.141, 0.141) | 0.034 (-0.105, 0.174) |
| Expressive Suppression × Training Version (Inconsistent) | -0.011 (-0.215, 0.193) | -0.13 (-0.332, 0.072) |
| Expressive Suppression × Condition (Stress) | 0.034 (-0.168, 0.236) | -0.003 (-0.203, 0.197) |
| Expressive Suppression × Training Version (Inconsistent) × Condition (Stress) | -0.172 (-0.481, 0.137) | 0.038 (-0.268, 0.343) |
| BAS Reward Responsiveness | -0.028 (-0.229, 0.174) | 0.058 (-0.141, 0.257) |
| BAS Reward Responsiveness × Training Version (Inconsistent) | -0.011 (-0.28, 0.257) | -0.009 (-0.275, 0.256) |
| BAS Reward Responsiveness × Condition (Stress) | 0.034 (-0.226, 0.294) | 0.014 (-0.242, 0.271) |
| BAS Reward Responsiveness × Training Version (Inconsistent) × Condition (Stress) | 0.011 (-0.356, 0.378) | 0.117 (-0.245, 0.48) |
| BAS Fun Seeking | 0.14 (-0.005, 0.284) | -0.05 (-0.193, 0.093) |
| BAS Fun Seeking × Training Version (Inconsistent) | -0.025 (-0.247, 0.197) | -0.058 (-0.277, 0.161) |
| BAS Fun Seeking × Condition (Stress) | -0.105 (-0.341, 0.131) | -0.018 (-0.251, 0.215) |
| BAS Fun Seeking × Training Version (Inconsistent) × Condition (Stress) | -0.08 (-0.436, 0.276) | 0.047 (-0.304, 0.398) |
| BAS Drive | **-0.201 (-0.38, -0.022)*** | -0.055 (-0.232, 0.122) |
| BAS Drive × Training Version (Inconsistent) | 0.223 (-0.016, 0.462) | 0.068 (-0.167, 0.304) |
| BAS Drive × Condition (Stress) | **0.261 (0.014, 0.508)*** | 0.155 (-0.089, 0.399) |
| BAS Drive × Training Version (Inconsistent) × Condition (Stress) | -0.353 (-0.713, 0.007) | -0.267 (-0.622, 0.088) |
| BIS | -0.118 (-0.295, 0.059) | -0.014 (-0.186, 0.159) |
| BIS × Training Version (Inconsistent) | 0.174 (-0.097, 0.444) | **0.265 (0.001, 0.529)*** |
| BIS × Condition (Stress) | 0.074 (-0.145, 0.293) | 0.14 (-0.076, 0.357) |
| BIS × Training Version (Inconsistent) × Condition (Stress) | -0.138 (-0.474, 0.198) | -0.322 (-0.654, 0.009) |
| IUS | -0.043 (-0.205, 0.119) | -0.033 (-0.191, 0.125) |
| IUS × Training Version (Inconsistent) | -0.075 (-0.332, 0.181) | -0.184 (-0.433, 0.066) |
| IUS × Condition (Stress) | 0.189 (-0.057, 0.436) | -0.019 (-0.262, 0.224) |
| IUS × Training Version (Inconsistent) × Condition (Stress) | -0.006 (-0.374, 0.363) | **0.408 (0.044, 0.771)*** |
| STAI Trait Anxiety | -0.051 (-0.215, 0.113) | 0.009 (-0.15, 0.168) |
| STAI Trait Anxiety × Training Version (Inconsistent) | -0.043 (-0.298, 0.212) | -0.034 (-0.283, 0.214) |
| STAI Trait Anxiety × Condition (Stress) | -0.196 (-0.432, 0.04) | 0.137 (-0.096, 0.37) |
| STAI Trait Anxiety × Training Version (Inconsistent) × Condition (Stress) | 0.104 (-0.256, 0.465) | -0.151 (-0.507, 0.204) |
| Round × Training Version (Consistent) × BIS | 0.014 (-0.014, 0.042) | -0.012 (-0.036, 0.013) |
| Round × Training Version (Inconsistent) × BIS | 0.014 (-0.017, 0.045) | -0.012 (-0.039, 0.015) |
| Round × Training Version (Consistent) × IUS | -0.02 (-0.049, 0.009) | 0.004 (-0.021, 0.03) |
| Round × Training Version (Inconsistent) × IUS | **0.039 (0.003, 0.074)*** | 0.029 (-0.002, 0.059) |
| Round × Training Version (Consistent) × STAI Trait Anxiety | **0.031 (0.002, 0.06)*** | **-0.026 (-0.051, -0.001)*** |
| Round × Training Version (Inconsistent) × STAI Trait Anxiety | 0.001 (-0.034, 0.036) | -0.016 (-0.046, 0.015) |

Note: Standardized weights are reported alongside 95% confidence intervals in parentheses. * *p* < .05, ** *p* < .01, *** *p* < .001.

## Table S10. Linear mixed effects model results predicting Rounds 1–5 performance score and completion time (standardized relative to round 1 – consistent – control) from emotion-cognition traits and their interactions with Training Version, change in RMSSD relative to base, and Round.

| Predictor | Performance Score | Completion Time |
| --- | --- | --- |
| Training Version (Inconsistent) | -0.048 (-0.216, 0.119) | **0.272 (0.11, 0.434)**** |
| RMSSD Change | 0.052 (-0.086, 0.19) | 0.029 (-0.093, 0.151) |
| Training Version (Inconsistent) × RMSSD Change | -0.088 (-0.268, 0.092) | -0.034 (-0.193, 0.126) |
| Round | **0.181 (0.154, 0.209)***** | **-0.236 (-0.26, -0.212)***** |
| Training Version (Inconsistent) × Round | -0.04 (-0.08, 0) | -0.015 (-0.05, 0.02) |
| RMSSD Change × Round | -0.017 (-0.056, 0.022) | 0.021 (-0.014, 0.055) |
| Training Version (Inconsistent) × RMSSD Change × Round | 0.016 (-0.037, 0.069) | -0.006 (-0.052, 0.041) |
| Cognitive Reappraisal | -0.071 (-0.177, 0.036) | -0.073 (-0.179, 0.032) |
| Cognitive Reappraisal × Training Version (Inconsistent) | -0.049 (-0.209, 0.11) | 0.127 (-0.031, 0.285) |
| Cognitive Reappraisal × RMSSD Change | -0.015 (-0.133, 0.103) | 0.014 (-0.091, 0.12) |
| Cognitive Reappraisal × Training Version (Inconsistent) × RMSSD Change | -0.028 (-0.193, 0.136) | -0.054 (-0.199, 0.092) |
| Expressive Suppression | -0.022 (-0.125, 0.081) | 0.014 (-0.088, 0.116) |
| Expressive Suppression × Training Version (Inconsistent) | -0.054 (-0.208, 0.101) | -0.077 (-0.23, 0.077) |
| Expressive Suppression × RMSSD Change | -0.031 (-0.183, 0.121) | 0.018 (-0.118, 0.155) |
| Expressive Suppression × Training Version (Inconsistent) × RMSSD Change | 0.009 (-0.187, 0.204) | -0.009 (-0.184, 0.165) |
| BAS Reward Responsiveness | 0.004 (-0.122, 0.129) | 0.058 (-0.066, 0.183) |
| BAS Reward Responsiveness × Training Version (Inconsistent) | -0.025 (-0.202, 0.151) | 0.081 (-0.094, 0.257) |
| BAS Reward Responsiveness × RMSSD Change | -0.077 (-0.224, 0.071) | -0.087 (-0.219, 0.045) |
| BAS Reward Responsiveness × Training Version (Inconsistent) × RMSSD Change | 0.035 (-0.134, 0.204) | 0.061 (-0.089, 0.211) |
| BAS Fun Seeking | 0.085 (-0.028, 0.199) | -0.096 (-0.209, 0.018) |
| BAS Fun Seeking × Training Version (Inconsistent) | -0.062 (-0.23, 0.106) | -0.01 (-0.177, 0.157) |
| BAS Fun Seeking × RMSSD Change | 0.074 (-0.085, 0.234) | -0.114 (-0.257, 0.028) |
| BAS Fun Seeking × Training Version (Inconsistent) × RMSSD Change | 0.017 (-0.163, 0.196) | 0.15 (-0.009, 0.31) |
| BAS Drive | -0.08 (-0.203, 0.043) | 0.03 (-0.091, 0.152) |
| BAS Drive × Training Version (Inconsistent) | 0.053 (-0.123, 0.228) | -0.098 (-0.272, 0.076) |
| BAS Drive × RMSSD Change | 0.1 (-0.111, 0.312) | 0.158 (-0.032, 0.349) |
| BAS Drive × Training Version (Inconsistent) × RMSSD Change | -0.124 (-0.362, 0.114) | **-0.262 (-0.475, -0.048)*** |
| BIS | -0.054 (-0.18, 0.072) | 0.038 (-0.083, 0.159) |
| BIS × Training Version (Inconsistent) | 0.075 (-0.114, 0.264) | 0.101 (-0.082, 0.284) |
| BIS × RMSSD Change | 0.106 (-0.024, 0.236) | -0.075 (-0.192, 0.042) |
| BIS × Training Version (Inconsistent) × RMSSD Change | -0.137 (-0.281, 0.007) | 0.118 (-0.01, 0.247) |
| IUS | -0.03 (-0.165, 0.105) | -0.086 (-0.216, 0.045) |
| IUS × Training Version (Inconsistent) | -0.002 (-0.208, 0.204) | 0.048 (-0.151, 0.248) |
| IUS × RMSSD Change | -0.144 (-0.289, 0.001) | -0.042 (-0.173, 0.089) |
| IUS × Training Version (Inconsistent) × RMSSD Change | 0.112 (-0.077, 0.301) | -0.068 (-0.238, 0.101) |
| STAI Trait Anxiety | **-0.144 (-0.275, -0.013)*** | 0.087 (-0.04, 0.213) |
| STAI Trait Anxiety × Training Version (Inconsistent) | 0.023 (-0.176, 0.223) | -0.1 (-0.294, 0.093) |
| STAI Trait Anxiety × RMSSD Change | -0.075 (-0.197, 0.048) | 0.046 (-0.063, 0.155) |
| STAI Trait Anxiety × Training Version (Inconsistent) × RMSSD Change | 0.062 (-0.121, 0.244) | -0.046 (-0.207, 0.116) |
| Round × Training Version (Consistent) × BIS | 0.013 (-0.015, 0.042) | -0.008 (-0.033, 0.016) |
| Round × Training Version (Inconsistent) × BIS | 0.019 (-0.015, 0.052) | -0.007 (-0.036, 0.022) |
| Round × Training Version (Consistent) × IUS | -0.017 (-0.047, 0.012) | 0.007 (-0.019, 0.033) |
| Round × Training Version (Inconsistent) × IUS | **0.04 (0.003, 0.077)*** | **0.034 (0.002, 0.066)*** |
| Round × Training Version (Consistent) × STAI Trait Anxiety | **0.035 (0.006, 0.065)*** | **-0.029 (-0.054, -0.003)*** |
| Round × Training Version (Inconsistent) × STAI Trait Anxiety | -0.001 (-0.037, 0.034) | -0.019 (-0.05, 0.012) |

Note: Standardized weights are reported alongside 95% confidence intervals in parentheses. * *p* < .05, ** *p* < .01, *** *p* < .001.

## Table S11. Linear mixed effects model results predicting Rounds 1–5 performance score and completion time (standardized relative to round 1 – consistent – control) from emotion-cognition traits and their interactions with Training Version, change in MAP relative to base, and Round.

| Predictor | Performance Score | Completion Time |
| --- | --- | --- |
| Training Version (Inconsistent) | -0.044 (-0.21, 0.122) | **0.234 (0.081, 0.388)**** |
| MAP Change | 0.005 (-0.085, 0.095) | 0.024 (-0.054, 0.103) |
| Training Version (Inconsistent) × MAP Change | 0.056 (-0.082, 0.193) | 0.112 (-0.008, 0.231) |
| Round | **0.182 (0.154, 0.21)***** | **-0.239 (-0.263, -0.215)***** |
| Training Version (Inconsistent) × Round | -0.034 (-0.075, 0.006) | -0.008 (-0.043, 0.026) |
| MAP Change × Round | 0.005 (-0.024, 0.034) | 0.001 (-0.024, 0.026) |
| Training Version (Inconsistent) × MAP Change × Round | -0.025 (-0.07, 0.019) | -0.036 (-0.074, 0.002) |
| Cognitive Reappraisal | -0.056 (-0.158, 0.046) | -0.057 (-0.153, 0.039) |
| Cognitive Reappraisal × Training Version (Inconsistent) | -0.065 (-0.221, 0.091) | 0.096 (-0.05, 0.243) |
| Cognitive Reappraisal × MAP Change | -0.032 (-0.101, 0.037) | -0.019 (-0.08, 0.041) |
| Cognitive Reappraisal × Training Version (Inconsistent) × MAP Change | 0.018 (-0.099, 0.136) | 0.07 (-0.033, 0.173) |
| Expressive Suppression | -0.016 (-0.115, 0.083) | 0.012 (-0.081, 0.105) |
| Expressive Suppression × Training Version (Inconsistent) | -0.056 (-0.206, 0.094) | -0.098 (-0.239, 0.043) |
| Expressive Suppression × MAP Change | -0.001 (-0.061, 0.059) | -0.013 (-0.065, 0.039) |
| Expressive Suppression × Training Version (Inconsistent) × MAP Change | 0.015 (-0.082, 0.112) | 0.037 (-0.048, 0.121) |
| BAS Reward Responsiveness | 0.017 (-0.107, 0.141) | 0.091 (-0.026, 0.208) |
| BAS Reward Responsiveness × Training Version (Inconsistent) | -0.046 (-0.221, 0.129) | 0.022 (-0.143, 0.187) |
| BAS Reward Responsiveness × MAP Change | -0.041 (-0.128, 0.045) | 0.008 (-0.068, 0.084) |
| BAS Reward Responsiveness × Training Version (Inconsistent) × MAP Change | 0.102 (-0.021, 0.226) | -0.002 (-0.111, 0.107) |
| BAS Fun Seeking | 0.082 (-0.033, 0.196) | -0.09 (-0.198, 0.017) |
| BAS Fun Seeking × Training Version (Inconsistent) | -0.088 (-0.258, 0.081) | -0.064 (-0.224, 0.096) |
| BAS Fun Seeking × MAP Change | 0.003 (-0.07, 0.076) | 0 (-0.064, 0.065) |
| BAS Fun Seeking × Training Version (Inconsistent) × MAP Change | -0.062 (-0.176, 0.052) | -0.033 (-0.134, 0.067) |
| BAS Drive | -0.095 (-0.212, 0.021) | 0.015 (-0.095, 0.125) |
| BAS Drive × Training Version (Inconsistent) | 0.093 (-0.077, 0.262) | -0.036 (-0.196, 0.124) |
| BAS Drive × MAP Change | 0.043 (-0.017, 0.103) | 0.007 (-0.045, 0.059) |
| BAS Drive × Training Version (Inconsistent) × MAP Change | -0.04 (-0.147, 0.066) | 0.021 (-0.073, 0.114) |
| BIS | -0.059 (-0.178, 0.06) | 0.057 (-0.053, 0.167) |
| BIS × Training Version (Inconsistent) | 0.073 (-0.112, 0.258) | 0.045 (-0.126, 0.216) |
| BIS × MAP Change | 0.048 (-0.012, 0.108) | -0.026 (-0.079, 0.026) |
| BIS × Training Version (Inconsistent) × MAP Change | -0.07 (-0.174, 0.033) | 0.018 (-0.073, 0.108) |
| IUS | -0.054 (-0.187, 0.078) | -0.087 (-0.21, 0.035) |
| IUS × Training Version (Inconsistent) | 0.055 (-0.152, 0.262) | 0.035 (-0.156, 0.227) |
| IUS × MAP Change | -0.038 (-0.118, 0.043) | 0.039 (-0.032, 0.11) |
| IUS × Training Version (Inconsistent) × MAP Change | 0.074 (-0.056, 0.203) | -0.027 (-0.142, 0.087) |
| STAI Trait Anxiety | -0.103 (-0.231, 0.024) | 0.076 (-0.042, 0.194) |
| STAI Trait Anxiety × Training Version (Inconsistent) | -0.004 (-0.201, 0.192) | -0.049 (-0.231, 0.133) |
| STAI Trait Anxiety × MAP Change | -0.065 (-0.147, 0.018) | -0.058 (-0.131, 0.014) |
| STAI Trait Anxiety × Training Version (Inconsistent) × MAP Change | 0.114 (-0.013, 0.241) | 0.103 (-0.008, 0.215) |
| Round × Training Version (Consistent) × BIS | 0.012 (-0.017, 0.041) | -0.014 (-0.039, 0.011) |
| Round × Training Version (Inconsistent) × BIS | 0.019 (-0.013, 0.051) | -0.004 (-0.032, 0.023) |
| Round × Training Version (Consistent) × IUS | -0.016 (-0.046, 0.015) | 0.009 (-0.017, 0.035) |
| Round × Training Version (Inconsistent) × IUS | 0.035 (-0.002, 0.071) | 0.019 (-0.012, 0.05) |
| Round × Training Version (Consistent) × STAI Trait Anxiety | **0.03 (0.001, 0.06)*** | **-0.029 (-0.054, -0.003)*** |
| Round × Training Version (Inconsistent) × STAI Trait Anxiety | -0.002 (-0.038, 0.035) | -0.018 (-0.05, 0.013) |

Note: Standardized weights are reported alongside 95% confidence intervals in parentheses. * *p* < .05, ** *p* < .01, *** *p* < .001.

## Table S12. Linear mixed effects model results predicting Rounds 1–5 performance score and completion time (standardized relative to round 1 – consistent – control) from emotion-cognition traits and their interactions with Training Version, change in HR relative to base, and Round.

| Predictor | Performance Score | Completion Time |
| --- | --- | --- |
| Training Version (Inconsistent) | -0.013 (-0.184, 0.157) | **0.299 (0.131, 0.467)***** |
| HR Change | 0.099 (-0.008, 0.206) | 0.037 (-0.06, 0.134) |
| Training Version (Inconsistent) × HR Change | -0.119 (-0.265, 0.026) | -0.045 (-0.177, 0.087) |
| Round | **0.193 (0.164, 0.223)***** | **-0.238 (-0.264, -0.212)***** |
| Training Version (Inconsistent) × Round | **-0.053 (-0.095, -0.01)*** | -0.016 (-0.054, 0.021) |
| HR Change × Round | -0.021 (-0.051, 0.009) | -0.026 (-0.052, 0) |
| Training Version (Inconsistent) × HR Change × Round | 0.025 (-0.018, 0.069) | 0.015 (-0.022, 0.053) |
| Cognitive Reappraisal | -0.056 (-0.158, 0.046) | -0.067 (-0.171, 0.037) |
| Cognitive Reappraisal × Training Version (Inconsistent) | -0.062 (-0.218, 0.094) | 0.111 (-0.048, 0.27) |
| Cognitive Reappraisal × HR Change | -0.023 (-0.098, 0.052) | 0.008 (-0.061, 0.078) |
| Cognitive Reappraisal × Training Version (Inconsistent) × HR Change | -0.002 (-0.111, 0.108) | 0.013 (-0.088, 0.113) |
| Expressive Suppression | -0.015 (-0.117, 0.086) | 0.017 (-0.087, 0.12) |
| Expressive Suppression × Training Version (Inconsistent) | -0.056 (-0.21, 0.097) | -0.109 (-0.265, 0.048) |
| Expressive Suppression × HR Change | 0.027 (-0.049, 0.103) | 0.007 (-0.063, 0.077) |
| Expressive Suppression × Training Version (Inconsistent) × HR Change | -0.047 (-0.16, 0.067) | 0.004 (-0.1, 0.108) |
| BAS Reward Responsiveness | -0.009 (-0.135, 0.117) | 0.08 (-0.048, 0.208) |
| BAS Reward Responsiveness × Training Version (Inconsistent) | -0.021 (-0.197, 0.155) | 0.042 (-0.137, 0.221) |
| BAS Reward Responsiveness × HR Change | 0.033 (-0.058, 0.123) | 0.04 (-0.043, 0.123) |
| BAS Reward Responsiveness × Training Version (Inconsistent) × HR Change | -0.001 (-0.132, 0.131) | 0.018 (-0.102, 0.138) |
| BAS Fun Seeking | 0.101 (-0.011, 0.213) | -0.085 (-0.199, 0.029) |
| BAS Fun Seeking × Training Version (Inconsistent) | -0.099 (-0.266, 0.069) | 0.012 (-0.158, 0.182) |
| BAS Fun Seeking × HR Change | -0.012 (-0.093, 0.069) | -0.034 (-0.109, 0.041) |
| BAS Fun Seeking × Training Version (Inconsistent) × HR Change | 0.024 (-0.104, 0.152) | -0.101 (-0.218, 0.016) |
| BAS Drive | -0.086 (-0.204, 0.033) | 0.01 (-0.11, 0.131) |
| BAS Drive × Training Version (Inconsistent) | 0.066 (-0.104, 0.236) | -0.043 (-0.216, 0.131) |
| BAS Drive × HR Change | 0.047 (-0.044, 0.139) | 0.016 (-0.069, 0.1) |
| BAS Drive × Training Version (Inconsistent) × HR Change | -0.051 (-0.179, 0.078) | -0.014 (-0.132, 0.105) |
| BIS | -0.107 (-0.232, 0.017) | 0.057 (-0.066, 0.18) |
| BIS × Training Version (Inconsistent) | 0.167 (-0.026, 0.36) | 0.103 (-0.088, 0.293) |
| BIS × HR Change | 0.065 (-0.018, 0.148) | 0.001 (-0.078, 0.079) |
| BIS × Training Version (Inconsistent) × HR Change | -0.111 (-0.248, 0.026) | -0.03 (-0.157, 0.096) |
| IUS | -0.026 (-0.157, 0.104) | -0.064 (-0.194, 0.065) |
| IUS × Training Version (Inconsistent) | -0.052 (-0.26, 0.156) | 0.03 (-0.176, 0.235) |
| IUS × HR Change | 0.079 (-0.009, 0.168) | -0.036 (-0.118, 0.045) |
| IUS × Training Version (Inconsistent) × HR Change | 0.02 (-0.123, 0.163) | 0.053 (-0.079, 0.185) |
| STAI Trait Anxiety | -0.066 (-0.195, 0.063) | 0.078 (-0.05, 0.206) |
| STAI Trait Anxiety × Training Version (Inconsistent) | -0.045 (-0.247, 0.157) | -0.095 (-0.294, 0.104) |
| STAI Trait Anxiety × HR Change | -0.065 (-0.15, 0.021) | 0.031 (-0.05, 0.111) |
| STAI Trait Anxiety × Training Version (Inconsistent) × HR Change | 0.07 (-0.066, 0.205) | -0.001 (-0.127, 0.125) |
| Round × Training Version (Consistent) × BIS | 0.026 (-0.006, 0.057) | -0.016 (-0.043, 0.012) |
| Round × Training Version (Inconsistent) × BIS | 0.009 (-0.028, 0.046) | -0.024 (-0.056, 0.009) |
| Round × Training Version (Consistent) × IUS | -0.007 (-0.038, 0.024) | 0.004 (-0.023, 0.031) |
| Round × Training Version (Inconsistent) × IUS | **0.053 (0.014, 0.093)**** | **0.039 (0.005, 0.074)*** |
| Round × Training Version (Consistent) × STAI Trait Anxiety | 0.015 (-0.017, 0.047) | -0.027 (-0.055, 0.001) |
| Round × Training Version (Inconsistent) × STAI Trait Anxiety | 0.002 (-0.036, 0.04) | -0.011 (-0.044, 0.022) |

Note: Standardized weights are reported alongside 95% confidence intervals in parentheses. * *p* < .05, ** *p* < .01, *** *p* < .001.

## Table S13. Linear mixed effects model results predicting Rounds 5–6 performance score and completion time (standardized relative to round 1 – consistent – control) from emotion-cognition traits and their interactions with Training Version, Stress Condition, Round, and Round 6 Version Switch.

| Predictor | Performance Score | Completion Time |
| --- | --- | --- |
| Training Version (Inconsistent) | -0.137 (-0.487, 0.213) | **0.268 (0.037, 0.498)*** |
| Condition (Stress) | 0.129 (-0.237, 0.495) | 0.084 (-0.154, 0.322) |
| Switch | -0.071 (-0.388, 0.245) | -0.06 (-0.268, 0.148) |
| Training Version (Inconsistent) × Condition (Stress) | -0.146 (-0.67, 0.378) | -0.166 (-0.509, 0.176) |
| Training Version (Inconsistent) × Switch | 0.063 (-0.424, 0.55) | 0.057 (-0.264, 0.377) |
| Condition (Stress) × Switch | 0.256 (-0.23, 0.741) | -0.021 (-0.338, 0.295) |
| Training Version (Inconsistent) × Condition (Stress) × Switch | -0.336 (-1.044, 0.372) | 0.094 (-0.37, 0.557) |
| Round | 0.127 (-0.074, 0.327) | **-0.102 (-0.203, 0)*** |
| Training Version (Inconsistent) × Round | 0.039 (-0.276, 0.354) | 0.007 (-0.152, 0.167) |
| Condition (Stress) × Round | -0.105 (-0.442, 0.232) | 0.007 (-0.165, 0.179) |
| Switch × Round | **-0.435 (-0.719, -0.15)**** | **0.226 (0.082, 0.371)**** |
| Training Version (Inconsistent) × Condition (Stress) × Round | 0.074 (-0.403, 0.551) | 0.053 (-0.189, 0.296) |
| Training Version (Inconsistent) × Switch × Round | 0.411 (-0.029, 0.852) | **-0.342 (-0.565, -0.119)**** |
| Condition (Stress) × Switch × Round | -0.074 (-0.522, 0.373) | 0.015 (-0.213, 0.243) |
| Training Version (Inconsistent) × Condition (Stress) × Switch × Round | 0.272 (-0.377, 0.922) | -0.116 (-0.446, 0.214) |
| Cognitive Reappraisal | 0.052 (-0.233, 0.336) | 0.015 (-0.172, 0.203) |
| Cognitive Reappraisal × Training Version (Inconsistent) | -0.28 (-0.656, 0.096) | -0.107 (-0.355, 0.141) |
| Cognitive Reappraisal × Condition (Stress) | -0.038 (-0.466, 0.391) | 0.098 (-0.182, 0.378) |
| Cognitive Reappraisal × Switch | **-0.578 (-0.984, -0.171)**** | -0.258 (-0.526, 0.01) |
| Cognitive Reappraisal × Training Version (Inconsistent) × Condition (Stress) | 0.087 (-0.514, 0.687) | 0.054 (-0.336, 0.445) |
| Cognitive Reappraisal × Training Version (Inconsistent) × Switch | **0.62 (0.054, 1.186)*** | **0.392 (0.021, 0.763)*** |
| Cognitive Reappraisal × Condition (Stress) × Switch | 0.477 (-0.087, 1.041) | 0.146 (-0.224, 0.515) |
| Cognitive Reappraisal × Training Version (Inconsistent) × Condition (Stress) × Switch | -0.236 (-1.045, 0.573) | -0.245 (-0.772, 0.282) |
| Cognitive Reappraisal × Round | 0.126 (-0.131, 0.382) | 0.017 (-0.112, 0.147) |
| Cognitive Reappraisal × Training Version (Inconsistent) × Round | -0.102 (-0.441, 0.237) | -0.026 (-0.197, 0.146) |
| Cognitive Reappraisal × Condition (Stress) × Round | -0.187 (-0.585, 0.21) | -0.075 (-0.277, 0.128) |
| Cognitive Reappraisal × Switch × Round | 0.048 (-0.318, 0.414) | -0.099 (-0.284, 0.086) |
| Cognitive Reappraisal × Training Version (Inconsistent) × Condition (Stress) × Round | 0.288 (-0.264, 0.841) | 0.054 (-0.228, 0.336) |
| Cognitive Reappraisal × Training Version (Inconsistent) × Switch × Round | -0.106 (-0.621, 0.41) | 0.089 (-0.173, 0.35) |
| Cognitive Reappraisal × Condition (Stress) × Switch × Round | 0.005 (-0.512, 0.523) | 0.118 (-0.146, 0.381) |
| Cognitive Reappraisal × Training Version (Inconsistent) × Condition (Stress) × Switch × Round | -0.148 (-0.893, 0.598) | -0.1 (-0.48, 0.279) |
| Expressive Suppression | 0.076 (-0.149, 0.3) | -0.034 (-0.182, 0.114) |
| Expressive Suppression × Training Version (Inconsistent) | -0.039 (-0.367, 0.289) | 0.05 (-0.166, 0.266) |
| Expressive Suppression × Condition (Stress) | -0.035 (-0.379, 0.308) | 0.073 (-0.151, 0.297) |
| Expressive Suppression × Switch | -0.077 (-0.421, 0.267) | -0.027 (-0.254, 0.199) |
| Expressive Suppression × Training Version (Inconsistent) × Condition (Stress) | -0.13 (-0.68, 0.421) | -0.04 (-0.398, 0.318) |
| Expressive Suppression × Training Version (Inconsistent) × Switch | 0.126 (-0.372, 0.623) | -0.105 (-0.431, 0.222) |
| Expressive Suppression × Condition (Stress) × Switch | 0.108 (-0.394, 0.611) | -0.018 (-0.347, 0.311) |
| Expressive Suppression × Training Version (Inconsistent) × Condition (Stress) × Switch | -0.092 (-0.857, 0.673) | -0.062 (-0.562, 0.438) |
| Expressive Suppression × Round | -0.082 (-0.284, 0.12) | 0.015 (-0.087, 0.117) |
| Expressive Suppression × Training Version (Inconsistent) × Round | 0.064 (-0.231, 0.359) | -0.011 (-0.161, 0.138) |
| Expressive Suppression × Condition (Stress) × Round | 0.151 (-0.171, 0.472) | 0.009 (-0.156, 0.174) |
| Expressive Suppression × Switch × Round | -0.083 (-0.393, 0.226) | -0.041 (-0.198, 0.115) |
| Expressive Suppression × Training Version (Inconsistent) × Condition (Stress) × Round | 0.016 (-0.49, 0.522) | -0.007 (-0.265, 0.251) |
| Expressive Suppression × Training Version (Inconsistent) × Switch × Round | 0.066 (-0.384, 0.517) | 0.038 (-0.191, 0.266) |
| Expressive Suppression × Condition (Stress) × Switch × Round | 0.054 (-0.407, 0.516) | 0.035 (-0.199, 0.27) |
| Expressive Suppression × Training Version (Inconsistent) × Condition (Stress) × Switch × Round | -0.551 (-1.256, 0.154) | 0.008 (-0.351, 0.367) |
| BAS Reward Responsiveness | -0.069 (-0.382, 0.244) | 0.186 (-0.02, 0.393) |
| BAS Reward Responsiveness × Training Version (Inconsistent) | 0.067 (-0.401, 0.536) | -0.043 (-0.352, 0.266) |
| BAS Reward Responsiveness × Condition (Stress) | 0.058 (-0.391, 0.506) | -0.288 (-0.581, 0.005) |
| BAS Reward Responsiveness × Switch | 0.211 (-0.259, 0.682) | **-0.339 (-0.649, -0.029)*** |
| BAS Reward Responsiveness × Training Version (Inconsistent) × Condition (Stress) | -0.162 (-0.823, 0.5) | 0.268 (-0.164, 0.7) |
| BAS Reward Responsiveness × Training Version (Inconsistent) × Switch | -0.302 (-0.957, 0.353) | 0.308 (-0.121, 0.737) |
| BAS Reward Responsiveness × Condition (Stress) × Switch | -0.098 (-0.719, 0.523) | **0.535 (0.129, 0.942)*** |
| BAS Reward Responsiveness × Training Version (Inconsistent) × Condition (Stress) × Switch | 0.755 (-0.138, 1.648) | -0.508 (-1.091, 0.075) |
| BAS Reward Responsiveness × Round | 0.036 (-0.246, 0.318) | -0.078 (-0.22, 0.065) |
| BAS Reward Responsiveness × Training Version (Inconsistent) × Round | 0.023 (-0.399, 0.445) | 0.04 (-0.173, 0.254) |
| BAS Reward Responsiveness × Condition (Stress) × Round | 0.178 (-0.231, 0.587) | 0.156 (-0.052, 0.364) |
| BAS Reward Responsiveness × Switch × Round | -0.177 (-0.601, 0.246) | 0.09 (-0.125, 0.304) |
| BAS Reward Responsiveness × Training Version (Inconsistent) × Condition (Stress) × Round | -0.358 (-0.958, 0.243) | -0.049 (-0.354, 0.256) |
| BAS Reward Responsiveness × Training Version (Inconsistent) × Switch × Round | 0.063 (-0.53, 0.656) | -0.14 (-0.441, 0.161) |
| BAS Reward Responsiveness × Condition (Stress) × Switch × Round | -0.063 (-0.627, 0.502) | -0.131 (-0.417, 0.156) |
| BAS Reward Responsiveness × Training Version (Inconsistent) × Condition (Stress) × Switch × Round | -0.094 (-0.906, 0.718) | 0.081 (-0.331, 0.493) |
| BAS Fun Seeking | 0.133 (-0.117, 0.383) | -0.038 (-0.203, 0.127) |
| BAS Fun Seeking × Training Version (Inconsistent) | -0.146 (-0.498, 0.206) | 0.01 (-0.222, 0.241) |
| BAS Fun Seeking × Condition (Stress) | -0.075 (-0.45, 0.299) | 0.042 (-0.204, 0.288) |
| BAS Fun Seeking × Switch | -0.122 (-0.496, 0.251) | 0.095 (-0.151, 0.341) |
| BAS Fun Seeking × Training Version (Inconsistent) × Condition (Stress) | 0.08 (-0.567, 0.726) | -0.049 (-0.469, 0.371) |
| BAS Fun Seeking × Training Version (Inconsistent) × Switch | 0.449 (-0.139, 1.037) | -0.272 (-0.655, 0.111) |
| BAS Fun Seeking × Condition (Stress) × Switch | 0.195 (-0.395, 0.784) | 0.023 (-0.364, 0.41) |
| BAS Fun Seeking × Training Version (Inconsistent) × Condition (Stress) × Switch | -0.917 (-1.866, 0.032) | 0.148 (-0.468, 0.765) |
| BAS Fun Seeking × Round | -0.107 (-0.332, 0.119) | 0.017 (-0.097, 0.131) |
| BAS Fun Seeking × Training Version (Inconsistent) × Round | 0.102 (-0.214, 0.419) | -0.028 (-0.188, 0.133) |
| BAS Fun Seeking × Condition (Stress) × Round | 0.209 (-0.161, 0.578) | -0.027 (-0.216, 0.162) |
| BAS Fun Seeking × Switch × Round | 0.233 (-0.103, 0.569) | 0.01 (-0.16, 0.18) |
| BAS Fun Seeking × Training Version (Inconsistent) × Condition (Stress) × Round | -0.156 (-0.762, 0.45) | 0.047 (-0.262, 0.356) |
| BAS Fun Seeking × Training Version (Inconsistent) × Switch × Round | **-0.617 (-1.157, -0.077)*** | 0.131 (-0.143, 0.406) |
| BAS Fun Seeking × Condition (Stress) × Switch × Round | **-0.654 (-1.206, -0.102)*** | -0.101 (-0.382, 0.18) |
| BAS Fun Seeking × Training Version (Inconsistent) × Condition (Stress) × Switch × Round | **1.571 (0.691, 2.45)***** | -0.128 (-0.576, 0.321) |
| BAS Drive | 0.164 (-0.217, 0.545) | -0.077 (-0.328, 0.174) |
| BAS Drive × Training Version (Inconsistent) | 0.125 (-0.349, 0.599) | 0.124 (-0.188, 0.437) |
| BAS Drive × Condition (Stress) | -0.274 (-0.788, 0.24) | 0.241 (-0.095, 0.577) |
| BAS Drive × Switch | -0.378 (-0.874, 0.118) | 0.154 (-0.173, 0.48) |
| BAS Drive × Training Version (Inconsistent) × Condition (Stress) | 0.128 (-0.578, 0.834) | -0.259 (-0.721, 0.202) |
| BAS Drive × Training Version (Inconsistent) × Switch | -0.02 (-0.662, 0.622) | -0.266 (-0.686, 0.154) |
| BAS Drive × Condition (Stress) × Switch | 0.644 (-0.026, 1.314) | -0.386 (-0.824, 0.053) |
| BAS Drive × Training Version (Inconsistent) × Condition (Stress) × Switch | -0.638 (-1.575, 0.299) | 0.32 (-0.292, 0.931) |
| BAS Drive × Round | -0.325 (-0.668, 0.018) | 0.097 (-0.076, 0.271) |
| BAS Drive × Training Version (Inconsistent) × Round | 0.113 (-0.314, 0.539) | -0.131 (-0.347, 0.085) |
| BAS Drive × Condition (Stress) × Round | 0.261 (-0.225, 0.748) | -0.148 (-0.396, 0.1) |
| BAS Drive × Switch × Round | 0.44 (-0.007, 0.887) | -0.148 (-0.374, 0.078) |
| BAS Drive × Training Version (Inconsistent) × Condition (Stress) × Round | -0.136 (-0.79, 0.518) | 0.113 (-0.219, 0.446) |
| BAS Drive × Training Version (Inconsistent) × Switch × Round | -0.144 (-0.726, 0.438) | 0.204 (-0.092, 0.499) |
| BAS Drive × Condition (Stress) × Switch × Round | -0.351 (-0.974, 0.271) | 0.317 (0, 0.634) |
| BAS Drive × Training Version (Inconsistent) × Condition (Stress) × Switch × Round | -0.044 (-0.908, 0.82) | -0.247 (-0.686, 0.193) |
| BIS | -0.153 (-0.407, 0.1) | 0.091 (-0.076, 0.258) |
| BIS × Training Version (Inconsistent) | **0.49 (0.066, 0.915)*** | 0.199 (-0.081, 0.479) |
| BIS × Condition (Stress) | 0.173 (-0.21, 0.556) | 0.126 (-0.124, 0.375) |
| BIS × Switch | 0.387 (-0.023, 0.796) | -0.247 (-0.517, 0.022) |
| BIS × Training Version (Inconsistent) × Condition (Stress) | -0.501 (-1.14, 0.138) | **-0.421 (-0.837, -0.004)*** |
| BIS × Training Version (Inconsistent) × Switch | **-0.777 (-1.42, -0.135)*** | 0.067 (-0.355, 0.488) |
| BIS × Condition (Stress) × Switch | -0.329 (-0.87, 0.212) | 0.057 (-0.298, 0.412) |
| BIS × Training Version (Inconsistent) × Condition (Stress) × Switch | **0.942 (0.074, 1.81)*** | 0.195 (-0.372, 0.762) |
| BIS × Round | 0.185 (-0.043, 0.413) | -0.03 (-0.146, 0.085) |
| BIS × Training Version (Inconsistent) × Round | -0.333 (-0.715, 0.05) | -0.141 (-0.335, 0.052) |
| BIS × Condition (Stress) × Round | -0.313 (-0.662, 0.037) | -0.063 (-0.241, 0.114) |
| BIS × Switch × Round | **-0.402 (-0.77, -0.033)*** | 0.103 (-0.083, 0.29) |
| BIS × Training Version (Inconsistent) × Condition (Stress) × Round | **0.599 (0.018, 1.18)*** | 0.28 (-0.015, 0.575) |
| BIS × Training Version (Inconsistent) × Switch × Round | **0.755 (0.173, 1.337)*** | 0.054 (-0.241, 0.349) |
| BIS × Condition (Stress) × Switch × Round | 0.457 (-0.035, 0.948) | -0.073 (-0.322, 0.177) |
| BIS × Training Version (Inconsistent) × Condition (Stress) × Switch × Round | **-1.288 (-2.077, -0.498)**** | -0.171 (-0.572, 0.23) |
| IUS | -0.233 (-0.546, 0.079) | -0.043 (-0.25, 0.163) |
| IUS × Training Version (Inconsistent) | 0.152 (-0.274, 0.579) | -0.046 (-0.327, 0.235) |
| IUS × Condition (Stress) | 0.259 (-0.202, 0.72) | 0.043 (-0.258, 0.345) |
| IUS × Switch | -0.39 (-0.842, 0.063) | 0.003 (-0.295, 0.301) |
| IUS × Training Version (Inconsistent) × Condition (Stress) | 0.053 (-0.597, 0.703) | 0.245 (-0.178, 0.668) |
| IUS × Training Version (Inconsistent) × Switch | **0.648 (0.008, 1.288)*** | -0.007 (-0.426, 0.413) |
| IUS × Condition (Stress) × Switch | 0.244 (-0.415, 0.903) | -0.021 (-0.45, 0.408) |
| IUS × Training Version (Inconsistent) × Condition (Stress) × Switch | -0.655 (-1.593, 0.283) | 0.054 (-0.556, 0.664) |
| IUS × Round | 0.233 (-0.049, 0.514) | -0.048 (-0.19, 0.095) |
| IUS × Training Version (Inconsistent) × Round | -0.251 (-0.635, 0.133) | 0.001 (-0.193, 0.196) |
| IUS × Condition (Stress) × Round | -0.118 (-0.541, 0.304) | 0.038 (-0.177, 0.253) |
| IUS × Switch × Round | 0.066 (-0.341, 0.473) | -0.079 (-0.285, 0.127) |
| IUS × Training Version (Inconsistent) × Condition (Stress) × Round | -0.132 (-0.727, 0.462) | -0.065 (-0.367, 0.238) |
| IUS × Training Version (Inconsistent) × Switch × Round | -0.439 (-1.031, 0.152) | 0.165 (-0.135, 0.466) |
| IUS × Condition (Stress) × Switch × Round | -0.234 (-0.836, 0.369) | 0.004 (-0.303, 0.31) |
| IUS × Training Version (Inconsistent) × Condition (Stress) × Switch × Round | **1.101 (0.236, 1.965)*** | -0.122 (-0.562, 0.319) |
| STAI Trait Anxiety | 0.104 (-0.214, 0.421) | -0.035 (-0.244, 0.174) |
| STAI Trait Anxiety × Training Version (Inconsistent) | -0.274 (-0.718, 0.17) | -0.178 (-0.471, 0.115) |
| STAI Trait Anxiety × Condition (Stress) | -0.175 (-0.591, 0.241) | -0.116 (-0.388, 0.156) |
| STAI Trait Anxiety × Switch | -0.073 (-0.483, 0.337) | -0.002 (-0.272, 0.268) |
| STAI Trait Anxiety × Training Version (Inconsistent) × Condition (Stress) | 0.141 (-0.513, 0.796) | 0.22 (-0.204, 0.644) |
| STAI Trait Anxiety × Training Version (Inconsistent) × Switch | 0.239 (-0.357, 0.835) | 0.311 (-0.08, 0.702) |
| STAI Trait Anxiety × Condition (Stress) × Switch | 0.177 (-0.429, 0.784) | 0.29 (-0.101, 0.682) |
| STAI Trait Anxiety × Training Version (Inconsistent) × Condition (Stress) × Switch | -0.306 (-1.209, 0.597) | **-0.61 (-1.194, -0.027)*** |
| STAI Trait Anxiety × Round | -0.179 (-0.465, 0.107) | 0.024 (-0.121, 0.169) |
| STAI Trait Anxiety × Training Version (Inconsistent) × Round | 0.139 (-0.261, 0.539) | 0.086 (-0.116, 0.289) |
| STAI Trait Anxiety × Condition (Stress) × Round | 0.169 (-0.224, 0.563) | 0.075 (-0.126, 0.276) |
| STAI Trait Anxiety × Switch × Round | 0.246 (-0.123, 0.616) | -0.082 (-0.268, 0.105) |
| STAI Trait Anxiety × Training Version (Inconsistent) × Condition (Stress) × Round | 0.053 (-0.554, 0.66) | -0.173 (-0.483, 0.137) |
| STAI Trait Anxiety × Training Version (Inconsistent) × Switch × Round | -0.273 (-0.816, 0.27) | -0.079 (-0.355, 0.196) |
| STAI Trait Anxiety × Condition (Stress) × Switch × Round | -0.189 (-0.755, 0.377) | 0.102 (-0.187, 0.392) |
| STAI Trait Anxiety × Training Version (Inconsistent) × Condition (Stress) × Switch × Round | -0.079 (-0.915, 0.757) | 0.116 (-0.311, 0.543) |

Note: Standardized weights are reported alongside 95% confidence intervals in parentheses. * *p* < .05, ** *p* < .01, *** *p* < .001.
